# Immunomodulation of T Helper Cells by Tumor Microenvironment in Oral Cancer Is Associated With CCR8 Expression and Rapid Membrane Vitamin D Signaling Pathway

**DOI:** 10.3389/fimmu.2021.643298

**Published:** 2021-05-07

**Authors:** Marco Fraga, Milly Yáñez, Macarena Sherman, Faryd Llerena, Mauricio Hernandez, Guillermo Nourdin, Francisco Álvarez, Joaquín Urrizola, César Rivera, Liliana Lamperti, Lorena Nova, Silvia Castro, Omar Zambrano, Alejandro Cifuentes, León Campos, Sergio Moya, Juan Pastor, Marcelo Nuñez, Jorge Gatica, Jorge Figueroa, Felipe Zúñiga, Carlos Salomón, Gustavo Cerda, Ricardo Puentes, Gonzalo Labarca, Mabel Vidal, Reuben McGregor, Estefania Nova-Lamperti

**Affiliations:** ^1^ Molecular and Translational Immunology Laboratory, Clinical Biochemistry and Immunology Department, Pharmacy Faculty, Universidad de Concepción, Concepción, Chile; ^2^ Anatomy Pathology Unit and Dental Service, Oral Pathology Department, Hospital Las Higueras, Talcahuano, Chile; ^3^ Anatomy Pathology Unit, Hospital Guillermo Grant Benavente and Universidad de Concepción, Concepción, Chile; ^4^ Head and Neck Service, Hospital Guillermo Grant Benavente, Concepción, Chile; ^5^ Dental Service, Hospital Guillermo Grant Benavente, Concepción, Chile; ^6^ MELISA Institute, San Pedro de la Paz, Chile; ^7^ Oral Maxillofacial Surgery Department, Dental Faculty, Universidad San Sebastián, Concepción, Chile; ^8^ Department of Stomatology, Universidad de Talca, Talca, Chile; ^9^ PeveGen Laboratory, Concepción, Chile; ^10^ Centro de Salud Familiar (CESFAM) Penco Lirquén, Penco, Chile; ^11^ Surgery Service, Hospital Las Higueras, Talcahuano, Chile; ^12^ Dental Service, Maxillofacial Surgery Department, Hospital Las Higueras, Talcahuano, Chile; ^13^ Exosome Biology Laboratory, Centre for Clinical Diagnostics, UQ Centre for Clinical Research, Royal Brisbane and Women’s Hospital, Faculty of Medicine + Biomedical Sciences, The University of Queensland, Brisbane, QLD, Australia; ^14^ Advanced Microscopy Centre, Universidad de Concepción, Concepción, Chile; ^15^ Computer Science Department, Universidad de Concepción, Concepción, Chile; ^16^ Department of Molecular Medicine and Pathology, School of Medical Sciences, The University of Auckland, Auckland, New Zealand

**Keywords:** oral cancer, immunomodulation, cancer immunology, Th-like Tregs, CCR8

## Abstract

The immune system plays a key role in the protective response against oral cancer; however, the tumor microenvironment (TME) impairs this anti-cancer response by modulating T helper (Th) responses and promoting an anti-inflammatory environment. Regulatory T cells (Tregs) and Th2 effector cells (Teff) are associated with poor prognosis in oral squamous cell carcinoma (OSCC). However, the main immunomodulatory mechanisms associated with the enrichment of these subsets in OSCC remain unknown. We characterized Th-like lineages in Tregs and Teff and evaluated immunomodulatory changes induced by the TME in OSCC. Our phenotypic data revealed a higher distribution of tumour-infiltrating CCR8^+^ and Th2-like Treg in OSCC compared with non-malignant samples, whereas the percentages of Th1 cells were reduced in cancer. We then analyzed the direct effect of the TME by exposing T cell subsets to cancer secretomes and observed the OSCC secretome induced CCR8 expression and reduced cytokine production from both subsets. Transcriptomic analysis showed that the co-culture with OSCC secretome induced several gene changes associated with the vitamin D (VitD) signaling pathway in T cells. In addition, proteomic analysis identified the presence of several proteins associated with prostaglandin E2 (PGE2) production by rapid membrane VitD signaling and a reduced presence of the VitD binding protein. Thus, we analyzed the effect of VitD and PGE2 and observed that VitD promotes a regulatory Th2-like response with CCR8 expression whilst PGE2 also modulated CCR8 but inhibited cytokine production in combination with VitD. Finally, we evaluated the presence of CCR8 ligand in OSCC and observed increased chemokine CCL18, which was also able to upregulate CCR8 in activated Th cells. Overall, our data showed the immunomodulatory changes induced by the TME involving CCR8 expression and regulatory Th2 phenotypes, which are associated with PGE2 mediated VitD signaling pathway and CCL18 expression in OSCC.

## Introduction

Oral cancer is a malignant neoplasm developed in the oral cavity with high mortality and morbidity due to late-stage diagnosis and high incidence of metastasis (1). Oral squamous cell carcinoma (OSCC) is the most common type of oral cancer, representing more than 90% of the cases, and it has been linked with uncontrolled proliferation of squamous epithelial cells due to environmental-mediated genetic mutations. Risk factors such as long-term use of tobacco, alcohol abuse, excessive sun exposure, human papillomavirus (HPV) infection and a weakened immune system have been associated with OSCC (2, 3). In fact, it has been proposed that the origin of oral cancer is associated with DNA alteration mediated by environmental carcinogens, since 3 to 6 mutations are required to transform a healthy cell to a malignant cell (4). It is the impaired or overwhelmed anti-tumor immune response in the patient is the main factor that favors subsequent tumor progression (5). This altered response is not only associated with cancer cells escaping the immune control, but also to the immunomodulatory effects of the tumor microenvironment by contact dependent and soluble mechanisms, promoting a regulatory immune repertoire and inducing an anti-inflammatory environment.

The immunomodulatory mechanisms exerted by the tumor microenvironment include the contribution of cancer-associated immune cells, the expression of inhibitory checkpoints (6) and the production of soluble factors such as proteins, metabolites, chemical factors (7–9) and extracellular vesicles (10). In OSCC, the presence of cytokines such as IL-4, IL-5, IL-10, TGF-β, IL-17, IL-1α and immune-checkpoint inhibitors such as PD-L1 and Indoleamine 2,3-Dioxygenase 1 (IDO1) have been associated with poor prognosis (11). Several chemokines have also been associated with immunomodulation in OSCC such as CCL18, CXCL13 and CCL4. It is however not clear whether the chemokines exert direct changes in the repertoire or phenotype of immune cells. Moreover, high PD-L1 expression has been associated with good overall survival since its expression is higher in low-grade invasive OSCC cell lines than high-grade invasive OSCC cell lines (12, 13). Therefore, novel mechanisms need to be addressed to understand how this cancer modulates the immune system. In terms of metabolic changes, glycolysis-related proteins and mitochondrial enzymes (14), are also significantly increased in the carcinogenesis of OSCC making it is possible that the active glycolytic activity of cancer cells also affects the function of the immune cells. In terms of the immune repertoire, it has been shown that OSCC includes cells with a pro-tumoral role such as tumor associated macrophages (TAMs), cancer-associated fibroblasts (CAF) and regulatory T cells (Tregs) (11).

Previous data from our lab characterized the Th-like Tregs based on the expression of three chemokine receptors, immune transcriptomic profiles and specific lineage cytokine production, defining Th1 as CXCR3^+^CCR6^-^CCR4^+^, Th2 as CXCR3^-^CCR6^-^CCR4^+^, Th17 as CXCR3^-^CCR6^+^CCR4^+^ and Th1/17 CXCR3^+^CCR6^+^CCR4^+^ (15). We found that Th2-like Tregs expressed CCR8 and exhibited higher viability than other Th-like Tregs subsets, however suppression capacity was similar between subsets. However, Th2-like Tregs and Th2 Teff migrated more than other Th-like subsets a phenomenon not mediated by CCR4 expression. Finally, we analyzed the presence of Th-like Tregs in blood, thymus, spleen, liver, skin, colon and tissues and blood from patients with melanoma and colon cancer. We observed a high presence of Th2-like Tregs and Th2 effector cells (Teff) in melanoma and colorectal cancer at late-stage. Here we progress these findings by investigating the distribution of these subsets in a cancer that has been traditionally associated with late-stage detection to evaluate if there is a specific subset enriched in well-established tumors and the main mechanism associated with the enrichment of Th2 subsets in cancer areas.

In this study we analyzed the distribution of tissue resident Th-like Tregs and Teff in OSCC compared to non-malignant biopsies allowing us to investigate mechanisms associated with the presence of Th2-like Tregs in the tumor environment. Our results revealed that the Treg/Teff ratio and the percentages of Th2-like and CCR8^+^ T cell subsets were higher in OSCC biopsies compared to non-malignant biopsies. We then analyzed whether the OSCC tumor secreted-factors defined as secretome, were promoting these phenotypes and we observed that the OSCC secretome induced CCR8 expression and reduced cytokine production on both subsets. We then performed a proteomic and transcriptomic analysis of the secretome and the Th subsets after co-culture, and observed several proteins associated with prostaglandin E (PGE2) production by rapid membrane vitamin D (VitD) signaling and VitD transport in OSCC. In addition, several genes modulated by the OSCC secretome were associated with the VitD signaling pathway in both Th subsets. Since PGE2 and VitD have previously been related to CCR8 expression we analyzed their presence in the TME and their effect on T cell phenotype. The data revealed that cancer areas had higher PGE2 and the combination of the active form of VitD and PGE2 induced CCR8 in T cells and reduced cytokine production. In addition, Vitamin D promoted Th2-like Treg responses by regulating transcription factors and cytokine production. Finally, we evaluated the presence of CCR8 ligand in OSCC and observed higher chemokine CCL18, which was not promoting migration of CCR8^+^ cells but induced CCR8 expression by direct contact. Overall, our data suggest that the secretome from oral cancer induces CCR8 and promotes a Th2 lineage in the T cell repertoire by several mechanisms; rapid membrane VitD mediated PGE2 production, accumulation of Vitamin D in cancer areas and increasing CCL18 levels.

## Materials and Methods

### Patient

Peripheral blood and biopsies were obtained from healthy volunteers and patients, after informed consent was approved. Patients with and without OSCC were consented in accordance with the Talcahuano Health Service Research Ethics Committee, reference number 19-06-11 and Concepcion Health Service Research Ethics Committee, reference number 19-03-07 and in accordance with the Declaration of Helsinki. Patient data are described in **Table 1**.

**Table 1:** Patient data.

OSCC				
Patient ID	Gender	Age	Diagnosis	Stage
CO-01	Male	73	Well-differentiated squamous cell carcinoma	T2N1M0/II B
CO-02	Male	88	Well-differentiated squamous cell carcinoma	T2NOM0/II A
CO-03	Male	76	Moderately differentiated squamous cell carcinoma	T4N2M0/III A
CO-04	Male	56	Well-differentiated squamous cell carcinoma	T3N1M0/III A
CO-05	Male	70	Moderately differentiated squamous cell carcinoma	T2N2M0/III A
CO-011	Male	66	Moderately differentiated squamous cell carcinoma	T3 N2b MX/III A
CO-012	Male	73	Moderately differentiated squamous cell carcinoma	Unknown
CO-017	Male	74	Moderately differentiated squamous cell carcinoma	Unknown
CO-018	Male	66	Moderately differentiated squamous cell carcinoma	T1N0M0
CO-021	Male	58	Moderately differentiated squamous cell carcinoma	T4aN0M0/IIIB
CO-024	Female	76	Well-differentiated squamous cell carcinoma	T1N0M0/I
CO-025	Male	67	Well-differentiated squamous cell carcinoma	T2N0M0/II A
IHC-01	Male	76	Moderately differentiated squamous cell carcinoma	T3N2M0/III C
IHC-02	Male	70	Well-differentiated squamous cell carcinoma	T1N0M0/I
IHC-03	Male	74	Moderately differentiated squamous cell carcinoma	Unknown
**Control**				
**Patient ID**	**Gender**	**Age**	**Diagnosis**	
CO-06	Female	71	Conjunctival epithelial hyperplasia	
CO-07	Male	55	Conjunctival epithelial hyperplasia	
CO-08	Female	65	Conjunctival epithelial hyperplasia	
CO-09	Female	54	Conjunctival epithelial hyperplasia	
CO-010	Female	61	Conjunctival epithelial hyperplasia	
CO-013	Male	43	Healthy gum	
CO-015	Male	33	Healthy gum	
CO-016	Male	42	Fibrous hyperplasia	
CO-019	Female	67	Conjunctival epithelial hyperplasia	
CO-020	Female	50	Conjunctival epithelial hyperplasia	
CO-023	Female	30	Fibrous hyperplasia	
CO-026	Male	25	Healthy gum	
CO-027	Female	25	Healthy gum	
IHC-04	Female	72	Conjunctival epithelial hyperplasia	
IHC-05	Male	58	Conjunctival epithelial hyperplasia	
IHC-06	Male	72	Conjunctival epithelial hyperplasia	

### The Isolation of Th Subsets From Biopsies

Tissues from OSCC and control group were subjected to mechanical tissue disruption with sharps elements to reach small piece (< 0.1 cm). These pieces were then transferred to a recipient with serum-free medium X-VIVO15 (LONZA) with 1 mg/mL of collagenase (GIBCO) and 10 U/mL of DNase (Worthington) for an enzymatic digestion for 1 h at 37 °C under constant agitation. The digested sample was filtered (70um) to obtain cells from the biopsies. To obtain the mononuclear cell fraction, cells were isolated by density-gradient centrifugation at 400 x g for 20 min at room temperature using Lymphoprep (Axis Shield). Cells were washed with PBS at 300 x g for 10 min and live cells were counted using the viability Trypan Blue staining.

### Flow Cytometry

PBMCs and mononuclear cells obtained from tissues were stained with anti-CD4, anti-CD25, anti-CD127, anti-CXCR3, anti-CCR4, anti-CCR6, anti-CD45RA and anti-CCR8 for 30 min at 4°C in the dark. Samples were acquired on LSR Fortessa (BD) and files analyzed using FlowJo (Tree Star). Gates were set based on biological controls and fluorescence minus one control (FMO).

### Teff and Treg Cell Isolation From Peripheral Blood for Functional Assays

PBMCs were isolated as previously described and negative isolation of memory CD4^+^ T cells was performed with magnetic bead separation with the Memory CD4^+^ T Cell Isolation Kit, human (Miltenyi Biotec). Memory Teff and Tregs were then sorted on a BD FACSAria II (BD) based on CD4, CD25, CD127 and CD45RA expression.

### Secretome Collection

A standardized piece of tissue (weight about 0.1 g) from the oral cancer and control biopsies was cut and incubated in X-VIVO15 (LONZA) serum-free medium for 48 h at 37°C. After the incubation the medium was collected, debris was eliminated by centrifugation and filtration (0.22um), and the medium with all proteins and factors secreted from the tissue (Secretome) was stored by -80 °C until use.

### Cell Culture With Secretomes

Sorted Teff and Tregs from healthy donors were activated with anti-CD3/CD28 beads (1:5 ratio) (Life Technologies) and 1000 UI IL-2 for 5 days a 37°C. Then, 100 uL of OSCC and control secretomes were added to 2x10^5^ Teff or 2x10^5^ Tregs (in 100uL) in XVIVO-15 serum-free medium 48h a 37°C. After the incubation, the supernatants were stored for further cytokine production measurement using the Cytokine Bead Array Th1/2/17 Kit (BD) and the cells were counted (CountBright Absolute Counting Beads), stained with Live/Dead dye (Life Technologies), anti-CXCR3, anti-CCR4, anti-CCR6, anti-CCR8, anti-PD-1 and anti-TIGIT (all BioLegend) and analyzed by flow cytometry. For the analysis of cells after secretome co-culture, cells were washed after co-culture with secretome and cultured in new media X-VIVO15 (LONZA) serum-free medium for 48 h at 37°C with anti-CD3/CD28 beads (1:5 ratio) (Life Technologies) and 1000 UI IL-2. After the incubation, the supernatants were stored for further cytokine production measurement and the cells were stained with Live/Dead dye (Life Technologies), anti-CCR6, anti-CCR8, anti-PD-1 and anti-TIGIT (all BioLegend).

### RNA-Seq Targeted Panel

Sorted Teff and Tregs from healthy donors were activated with anti-CD3/CD28 beads (1:5 ratio) (Life Technologies) and 1000 UI IL-2 for 5 days a 37°C. Then, 100 uL of OSCC and control secretomes were added to 2x10^5^ Teff or 2x10^5^ Tregs (in 100uL) in XVIVO-15 serum-free medium 48 h a 37°C. Cells were lysed in TRIzol, and RNA was isolated with Direct-Zol RNA MicroPrep w/Zymo-Spin columns. RNA-seq was performed using the QIAGEN Human Inflammation and Immunity Transcriptome RNA targeted panel (QIAGEN). Samples were sequenced with the Illumina NextSeq using NextSeq 500/550 Mild Output Kit v2.5 (150 Cycles) (Illumina). Volcano plots and pathway analysis were performed initially using QIAseq targeted RNA data analysis tools (QIAGEN). In addition, the quality of each sequencing library was verified using FastQC software package and summarized using MultiQC software package (16). The reads were aligned to the human reference genome (hg38) using STAR (17), a high-performance community-standard aligner. The expected RSEM counts were rounded to the nearest integer value and the transcripts with zero counts across all the samples are filtered out. Differential expression analysis was performed using DESeq2 package (18) between the cohorts (OSCC versus Control, Teff OSCC versus Control and Treg OSCC versus Control). A pathway enrichment analysis was performed using the Gene Ontology Consortium database (data-version Released 2021-02-01) including biological processes. Cytoscape v.3.8.2 with the ClueGO plugin v.2.5.7 was used with a (p<0.01) and a kappa statistics score = 0.4 to calculate the relationships between the terms based on the similarity of their associated genes. P-value is the probability of seeing at least x number of genes out of the total n genes in the list annotated to a particular GO term, given the proportion of genes in the whole genome that are annotated to that GO Term.

### Proteomic Analysis

#### Secretome Protein Depletion

The secretome proteins were depleted with Top 2 Abundant Protein Depletion Spin Columns (Thermo Scientific), 200 ug of secretome proteins were added per column and the protocol suggested by the manufacturer was followed.

#### Protein Extraction and Digestion for nLC-MS/MS

The previously depleted proteins were subjected to precipitation using 5: 1 v/v cold acetone 100% v/v and incubated overnight at -20°C, then they were centrifuged at 15,000 g for 10 min, the supernatant was discarded, and the pellet was washed 3 times with acetone at 90% v/v, later the proteins were dried in a rotary concentrator at 4°C, and finally they were resuspended in 8 M urea with 25 mM of ammonium bicarbonate pH 8. The proteins were reduced using a final concentration of 20 mM DTT for 1 h, then they were alkylated incubating for 1 h with 20 mM iodoacetamide in the dark, then the proteins were quantified using the Qubit protein quantification kit. 10 ug of total proteins were diluted to 1 M urea using 25 mM ammonium bicarbonate pH 8, then the proteins were digested with trypsin/LyC (Promega) in a 1:50 ratio overnight at 37°C. The peptides were cleaned using Pierce C-18 Spin Columns (Thermo Scientific) using the protocol suggested by the manufacturer, the eluted peptides were dried using a rotary concentrator at 4°C and resuspended in 2% ACN with 0.1% v/v Formic Acid (MERCK), and quantified using Direct detect (MERCK Millipore).

#### Liquid Chromatography

200 ng of secretome tryptic peptides were injected in nanoELUTE (Bruker Daltonics, Bremen, Germany) ultra-high-pressure nano-flow chromatography system was coupled online to a hybrid trapped ion mobility spectrometry - quadrupole time of flight mass spectrometer (timsTOF Pro, Bruker Daltonics, Bremen, Germany) with a modified nano-electrospray ion source (CaptiveSpray, Bruker Daltonics). Liquid chromatography was performed at 50°C and with a constant flow of 400 nL/min on a reversed-phase column Aurora Series CSI (25 cm x 75µm i.d. C18 1.6 µm) (*ionopticks* Australia). Mobile phases A and B were watered with 0.1% formic acid (v/v) and 99.9/0.1% ACN/formic acid (v/vol), respectively. In 90-min experiments, peptides were separated with a linear gradient from 2 to 17% B within 57 min, followed by an increase to 25% B within 21 min and further to 35% within 13 min, followed by a washing step at 85% B and re-equilibration.

#### The timsTOF Pro Mass Spectrometer-The timsTOF Pro

All further experiments were acquired with a 100 ms ramp and 10 PASEF MS/MS scans per topN acquisition cycle. In TOF mass spectrometry, signal-to-noise ratios can conveniently be increased by summation of individual TOF scans. Thus, low-abundance precursors with an intensity below a ‘target value’ were repeatedly scheduled for PASEF-MS/MS scans until the summed ion count reached the target value (e.g. four times for a precursor with the intensity 5000 arbitrary units (a.u.) and a target value of 20,000 a.u.). The target value to 20,000 a.u was set. MS and MS/MS spectra were recorded from m/z 100 to 1700. Suitable precursor ions for PASEF-MS/MS were selected in real time from TIMS-MS survey scans by a sophisticated PASEF scheduling algorithm. A polygon filter was applied to the m/z and ion mobility plane to select features most likely representing peptide precursors rather than singly charged background ions. quadrupole isolation width was set to 2 Th for m/z < 700 and 3 Th for m/z > 700, and the collision energy was ramped stepwise as a function of increasing ion mobility: 52 eV for 0 –19% of the ramp time; 47 eV from 19 –38%; 42 eV from 38 –57%; 37 eV from 57–76%; and 32 eV for the remainder (19). The TIMS elution voltage was calibrated linearly to obtain reduced ion mobility coefficients (1/K0) using three selected ions of the Agilent ESI-L Tuning Mix (m/z 622, 922, 1222) (20). Collisional cross sections were calculated from the Mason Schamp equation.

#### Database Searching

Tandem mass spectra were extracted by Tims Control version 2.0. Charge state deconvolution and deisotoping were not performed. All MS/MS samples were analyzed using PEAKS Studio (Bioinformatics Solutions, Waterloo, ON Canada; version 10.5 (2019-11-20). PEAKS Studio was set up to search the [UniProt_SwissProt] database (unknown version, 21040 entries) assuming the digestion enzyme trypsin. PEAKS Studio was searched with a fragment ion mass tolerance of 0,050 Da and a parent ion tolerance of 50 PPM. Carbamidomethyl of cysteine was specified in PEAKS Studio as a fixed modification. Deamidated of asparagine and glutamine, oxidation of methionine, acetyl of the n-terminus and carbamyl of lysine and the n-terminus were specified in PEAKS Studio as variable modifications.

#### Criteria for Protein Identification

Scaffold (version Scaffold_4.8.9, Proteome Software Inc., Portland, OR) was used to validate MS/MS based peptide and protein identifications. Peptide identifications were accepted if they could be established at greater than 95,0% probability by the Peptide Prophet algorithm (21) with Scaffold delta-mass correction. Protein identifications were accepted if they could be established at greater than 99,0% probability and contained at least 2 identified peptides. Protein probabilities were assigned by the Protein Prophet algorithm (22). Proteins that contained similar peptides and could not be differentiated based on MS/MS analysis alone were grouped to satisfy the principles of parsimony.

#### Ingenuity Pathway Analysis (IPA) of Identified Proteins

Pathway enrichment analyses were performed with Ingenuity Pathway Analysis (IPA, Qiagen, Hilden, Germany) as previously described (23, 24). IPA was performed to identify canonical pathways, diseases and functions, and protein networks. Significantly enriched pathways for the proteins and pathways were identified with the criterion p-value < 0.05.

### Vitamin D in Secretomes

Levels of 25(OH)VitD in cancer and control secretomes were determined using the competitive imunoluminometric assay Maglumi 25-OH Vitamin D kit (Snibe) performed on the Maglumi fully auto analyzer according to manufacturer’s instructions.

### PGE2 ELISA

Levels of Prostaglandin E2 in cancer and control secretomes were determined by PGE2 high sensitivity ELISA kit (Enzo) according to manufacturer’s instructions.

### Vitamin D Effect on Th Differentiation

2x10^5^ sorted Teff (Treg-depleted) from healthy donors were activated with anti-CD3/CD28 beads (1:5 ratio) (Life Technologies) in XVIVO-15 media for 5 days a 37°C in the presence or absence of 1,25(OH)VitD (10nM in ethanol) or carrier (ethanol). The supernatants were stored for cytokine measurement using the Cytokine Bead Array Th1/2/17 Kit (BD) and the cells were counted (CountBright Absolute Counting Beads) and stained with Live/Dead dye (Life Technologies), anti-FOXP3, anti-GATA3, anti-Tbet and anti-ROR*γ*τ and analyzed by flow cytometry.

### CCR8 Upregulation in Vitamin D and Prostaglandin E2 Culture

Sorted Th cells from healthy donors were activated with anti-CD3/CD28 beads (1:5 ratio)(Life Technologies) in XVIVO-15 media for 5 days a 37°C. Then, prostaglandin E2 (10 uM), 1,25(OH)VitD (10nM) and recombinants chemokines CCL1 and CCL18 (0.5ug/mL) were added to 1x10^5^ Th in XVIVO-15 serum-free medium for 72h a 37°C. After the incubation, the supernatants were stored for further cytokine production measurement using the Cytokine Bead Array Th1/2/17 Kit (BD) and live cells were counted (CountBright Absolute Counting Beads), stained with anti-CCR8 and analyzed by flow cytometry.

### Immunohistochemistry

Control and OSCC tissue embedded in paraffin were cut into 10 um slides. Paraffin was then removed with alcohols in ascendant concentrations. Slides were incubated with primary antibody, rabbit pAb anti-CCL1 and anti-CCL18 (all Biorbyt), overnight at 4°C. After wash with PBS to eliminate the excess of primary antibody, the slides were incubated with secondary antibody (Donkey HRP anti-rabbit IgG) (Abcam) for 1h at room temperature. The excess of secondary antibody was removed with PBS, and the slides were revealed with diaminobenzidine and observed with an optical microscope. The semi quantification of CCL1 and CCL18 was performed using ImageJ as follows. Images were open and transform as RGB Stack (Image → Type), then stack montage were performed (Image → Stacks) and finally threshold was set up to identify the positive staining (Image → Adjust → Threshold). Finally, we set up measurements: Area, area fraction, limit to threshold and display label (Analyze → Set measurements) and measured the positive staining (Analyze → Measure).

### Chemotaxis Assays

T cell migration was assessed using a 96 well 5-μm-pore Transwell filter system (Corning). The top chambers were incubated with Cell Trace Violet^+^ memory Teffs and unstained memory Tregs, sorted and rested prior experiment. After resting, 5 × 10^4^ Teffs + 5 × 10^4^ Tregs in 50 uL X-VIVO15 serum-free medium were placed in the top chamber. The bottom chambers were filled with 100 uL X-VIVO15 serum-free only or 100 uL of X-VIVO15 with CCL18 (0.5 ug/mL, Novus Biologicals) or CCL1 (0.5 ug/mL, BioLegend). After 1h at 37°C, cells were harvested from bottom compartments and counted (CountBright Absolute Counting Beads) with flow cytometry. The percentage of migration for each subset was calculated as (number of Th cells in the bottom chamber after 60 min × 100)/initial number of Th cells in the top chamber.

### Statistical analysis

Statistical tests were performed using Prism 9 software (GraphPad). Data are expressed as mean ± SEM where applicable using individual values, column bar charts, box and whiskers plots. Unpaired t test was used to compare one variable between unpaired samples (control *vs* OSCC). Paired t test was used to compare one variable between paired samples (close *vs* distant). Two-way ANOVA was used to compare two related variables between subsets from the same donor (Th subsets). Ordinary One-way ANOVA was used to compare one related variable (CCL18 levels). Post hoc tests were used as indicated in the figure legends. p values are reported as follows: ^∗^p < 0.05, ^∗∗^p < 0.01, ^∗∗∗^p < 0.001, and ^∗∗∗∗^p < 0.0001.

## Results

### Th2-Like Tregs and CCR8^+^ Tregs Are Enriched in Biopsies From Patients With OSCC

**Figure 1:**
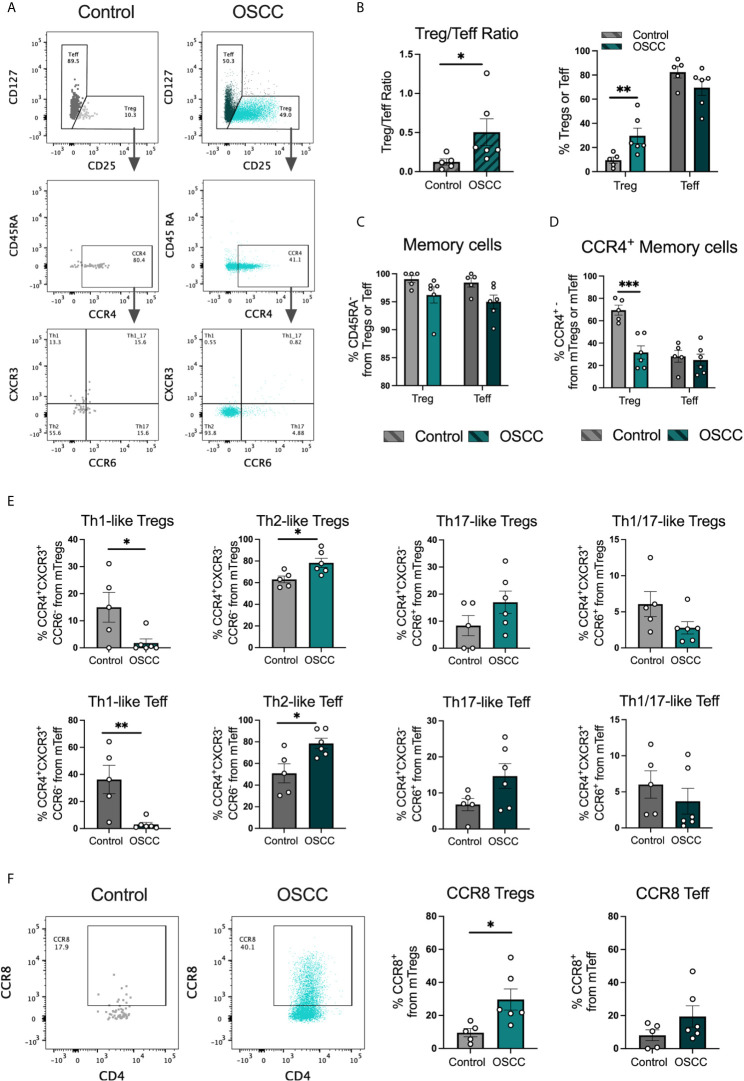
Th2-like T cell subsets and CCR8^+^ Tregs are the main tumor infiltrating Th subsets in OSCC. **(A)** Representative dot plots of tissue-resident cells obtained from a biopsy from a patient with OSCC and a control patient without malignancy. CD4^+^ T cells were divided into Teff and Tregs using CD25 and CD127 staining. Then, memory cells were selected as CD45RA^-^ and CCR4 and CCR8 expression was evaluated within the memory population. Within the CCR4^+^ subsets, Th1 were defined as CXCR3^+^CCR6^-^, Th2 as CXCR3^-^CCR6^-^, Th17 as CXCR3^-^CCR6^+^ and Th1/17 CXCR3^+^CCR6^+^. **(B)** Comparison of the Treg/Teff ratio and percentages of Tregs and Teff between OSCC patients and patients without malignancy. **(C)** Comparison of memory Tregs and Teff between OSCC patients and patients without malignancy. **(D)** Comparison of CCR4 expression within the memory Treg and Teff population between OSCC patients and patients without malignancy. **(E)** Comparison of tissue resident memory CCR4^+^ Th-like Tregs and Th-like Teff between OSCC patients and patients without malignancy. **(F)** Representative dot plots and comparison of CCR8 expression within the memory Treg and Teff population OSCC patients and patients without malignancy. Data are presented as mean ± SEM using bars with scatter dot plots (Unpaired t test). For all statistical tests, ^∗∗∗^p < 0.001, ^∗∗^p < 0.01 and ^∗^p < 0.05 were considered significant.

Peripheral blood derived Th-like Tregs and Teff have previously been characterized based on the expression of three chemokine receptors by our research group in several tissues including thymus, spleen, skin, colon and peripheral blood (15). In addition, we analyzed their distribution in malignant biopsies and observed a higher distribution of tissue-resident Th2-like subsets in melanoma and colorectal cancer compared to healthy skin and colon. In this study we analyzed the repertoire of infiltrated Th cells in oral cancer as this cancer is normally diagnosed at late stage. Tregs and Teff were identified by flow cytometry in tissues samples from patients with OSCC or patients without malignant oral lesions (**Table 1**) based on CD4, CD25, CD127 and CD45RA expression and chemokine receptors CCR4, CXCR3 and CCR6 expression (**Figure 1A**) as previously reported (15). FoxP3 staining was used to confirm Treg selection (**Supplementary Figure 1**). The Treg/Teff ratio between tissue resident T cells from patients with OSCC and their counterparts from donors without oral cancer was higher in the cancer, mostly due to an increase in Tregs (**Figure 1B**). Both Tregs and Teffs were mainly memory in the oral cavity with no difference observed between cancer and control (**Figure 1C**). From the memory population, we analyzed the expression of CCR4 and observed that Tregs in OSCC expressed lower CCR4 levels than tissue resident Tregs from controls, whereas no difference was observed in Teffs (**Figure 1D**). After analyzing the presence of CCR4 expression to define Th-like subsets, we analyzed the distribution of Th-like Treg and Teff subsets in both conditions. We observed increased percentages of Th2 and reduced percentages of Th1 subsets in Tregs and Teffs obtained from malignant tissues (**Figure 1E**). We have previously shown that Th2-like Tregs are the main CCR8^+^ population within Tregs, therefore we analyze the expression of this chemokine receptors in Tregs and Teff (**Figure 1F**). The analysis showed an increased expression of CCR8 in Tregs from OSCC samples in comparison with control samples and the presence of CCR8^+^ Tregs was independent of the presence of Th2-like Tregs. Our results were consistent with previous data in other malignancies, showing an imbalance between Th2/Th1 subsets in cancer with more than half of the Tregs found in oral cancer being either Th2-like or CCR8^+^ Tregs. The origin of these subsets is unknown so we next studied whether the local OSCC environment could induce this phenotype.

### Secretome From Oral Cancer Promotes the Expression of CCR8, PD-1 and TIGIT But Suppress Cytokine Production in Th Cells in OSCC

**Figure 2:**
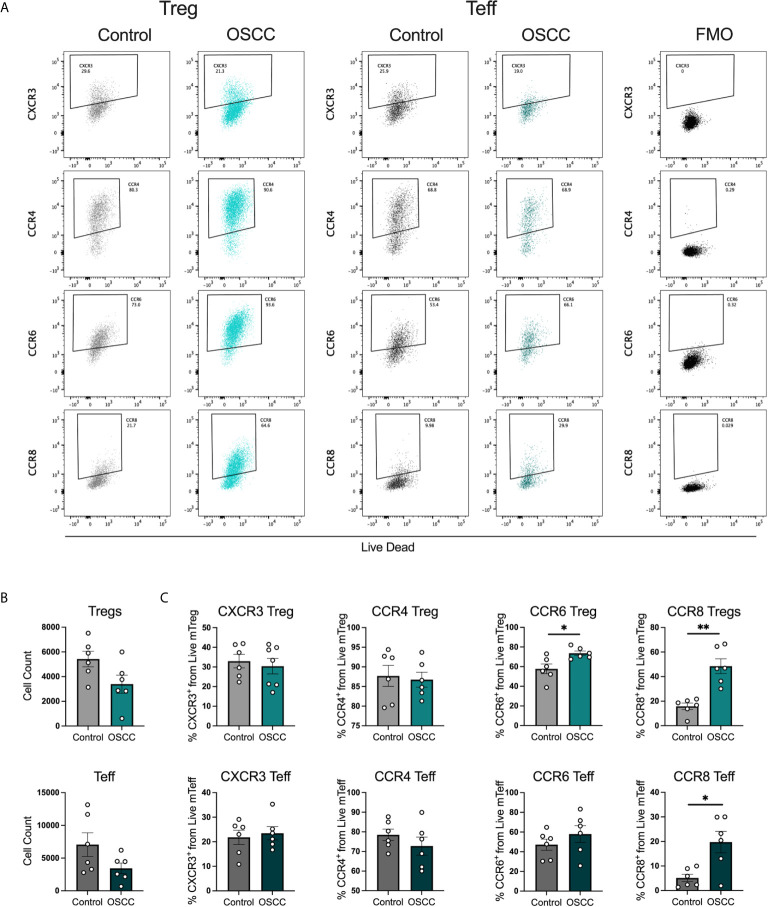
OSCC secretome up regulates CCR8 expression in Treg and Teff. **(A)** Representative dot plots of chemokine receptor expression CXCR3, CCR4, CCR6 and CCR8 in Tregs and Teff after co-culture with control or OSCC secretome. Briefly, sorted memory Tregs and Teff obtained from peripheral blood from 3 healthy donors were pre-activated with anti-CD3/CD28 beads (1:5) in the presence of IL-2 (1000U). After activation, 2x10^5^ Tregs and Teff were co-cultured with secretomes from OSCC or control samples for 48h. After co-culture, cells were stained with Live/Dead dye, chemokine receptor expression and counted with counting beads. **(B)** Comparison of cell counts between Tregs and Teff co-cultured with OSCC or control secretome. **(C)** Comparison of CXCR3, CCR4, CCR6 and CCR8 expression between Tregs and Teff co-cultured with OSCC or control secretome. Data are presented as mean ± SEM using bars with scatter dot plots (Unpaired t test). For all statistical tests, ^∗∗^p < 0.01 and ^∗^p < 0.05 were considered significant.

**Figure 3:**
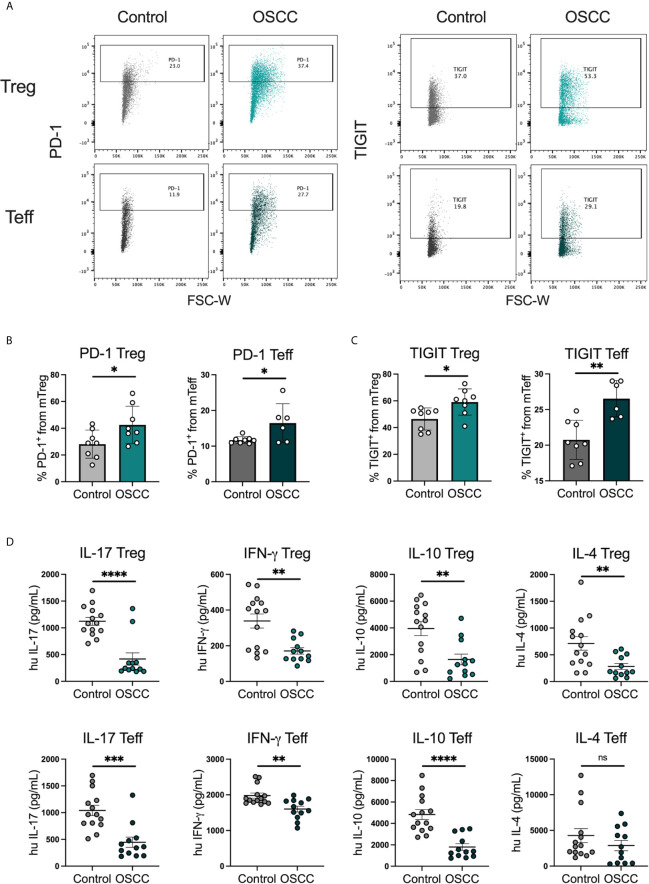
OSCC secretome promote PD-1 and TIGIT expression and inhibit cytokine production in comparison with control secretome. **(A)** Representative dot plots of PD-1 and TIGIT expression in Tregs and Teff after co-culture with control or OSCC secretome. Briefly, sorted memory Tregs and Teff obtained from peripheral blood from 4 healthy donors were pre-activated with anti-CD3/CD28 beads (1:5) in the presence of IL-2 (1000U). After activation, 2x10^5^ Tregs and Teff were co-cultured with secretomes from OSCC or control samples for 48h and cells were stained with PD-1 and TIGIT, whereas the supernatants were used to measure cytokines using cytokine bead array. Expression of both suppressive molecules was measured by flow cytometry. **(B)** Comparison of PD-1 expression between memory Tregs and Teff co-cultured with OSCC or control secretome. **(C)** Comparison of TIGIT expression between memory Tregs and Teff co-cultured with OSCC or control secretome. **(D)** Comparison of secreted Th cytokines from Tregs and Teff co-cultured with OSCC or control secretome. Data are presented as mean ± SEM using bars with scatter dot plots for phenotype and scatter dot plots for cytokine secretion (Unpaired t test). For all statistical tests, ^∗∗∗∗^p < 0.0001, ^∗∗∗^p < 0.001, ^∗∗^p < 0.01 and ^∗^p < 0.05 were considered significant. ns, not significant.

**Figure 4:**
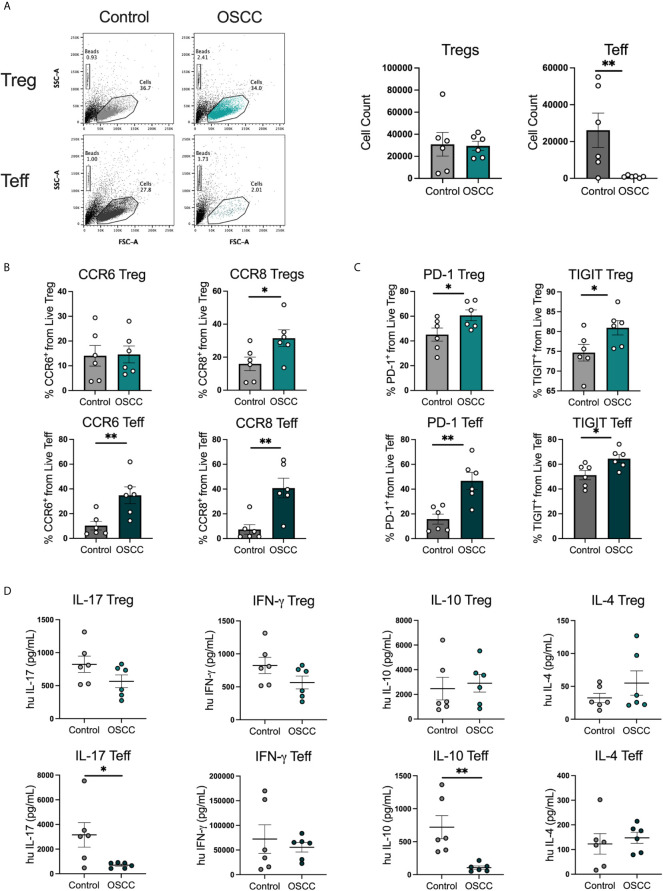
Pre-treatment with OSCC secretome affects Teff cell counts but CCR8, PD-1 and TIGIT expression is maintained after secretome removal in Treg and Teff. **(A)** Representative dot plots and cumulative data of Tregs and Teff cell counts after 48h of culture after removal of control or OSCC secretome. Briefly, sorted Tregs and Teff were pre-activated with anti-CD3/CD28 beads (1:5) in the presence of IL-2 (1000U). After activation, 1x10^5^ Tregs and Teff were co-cultured with secretomes from OSCC or control samples for 48h. Then, cells were washed and Tregs and Teff were cultured with anti-CD3/CD28 beads (1:5) in the presence of IL-2 (1000U) for 48h. **(B)** CCR6 and CCR8 expression was measured by flow cytometry in live Tregs and Teff. **(C)** PD-1 and TIGIT expression was measured by flow cytometry in live Tregs and Teff. **(D)** Supernatants of Tregs and Teff were collected and cytokines were measured with cytokine bead array. Data are presented as mean ± SEM using bars with scatter dot plots for phenotype and scatter dot plots for cytokine secretion (Unpaired t test). For all statistical tests, ^∗∗^p < 0.01 and ^∗^p < 0.05 were considered significant.

In order to identify whether the malignant environment was able to regulate the expression of CXCR3, CCR4, CCR6 and CCR8, we analyzed the direct effect of malignant and non-malignant secretome on viability and chemokine receptor expression in peripheral blood Tregs and Teff from healthy donors. The secretome has previously been defined as the proteins and metabolites secreted by a cell or tissue (25), thus we used a standard tissue piece of 0.1 g from a malignant or non-malignant biopsy to collect secretome in X-VIVO media without serum for 48h. Memory Tregs and Teff were activated and expanded for 5 days in the presence of IL-2 and anti-CD3CD28 beads. After expansion, cells were washed, co-cultured with malignant or control secretomes for 48h and expression of chemokine receptors was measured by flow cytometry (**Figure 2A**). First, we analyzed the cell count of live cells to see whether the co-culture with the secretome was affecting viability, however we observed a difference but it did not reach significance (**Figure 2B**). When expression of chemokine receptors was analyzed no difference in CXCR3 and CCR4 levels was observed for either subset, however in Tregs we observed a significant up regulation of CCR6. A significant increment was also observed in CCR8 expression within the Tregs and Teffs cultured with cancer secretome compared to control samples (**Figure 2C**), suggesting the tumor environment was regulating CCR8 expression in both subsets. Since the data showed a direct effect of the malignant environment on the T cell phenotype, we analyzed whether the secretome could also modulate the suppressive molecules PD-1 and TIGIT as well as cytokine secretion. PD-1 has been found expressed in cells with an exhausted phenotype (26) whereas TIGIT has been associated with selective Th1 and Th17, but not Th2 suppression (27), thus both molecules are relevant to cancer-related Th responses. Regarding the expression of PD-1 and TIGIT (**Figure 3A**), we observed that the co-culture between the T cell subsets and the malignant secretome induced PD-1 upregulation in Tregs and Teff in comparison with the control secretome (**Figure 3B**). Similar upregulation by malignant OSCC secretome was observed for TIGIT in both subsets (**Figure 3C**). Finally, when cytokines were analyzed, we observed that all cytokines were significantly inhibited in the presence of OSCC secretome except for IL-4 in Teffs (**Figure 3D**). Since CCR8 has been associated with a Th2 phenotype, we sorted CCR8^-^ and CCR8^+^ Tregs and Teff to evaluate the main cytokines produced by both subsets. Interestingly and similar to the data obtained from cancer secretomes, CCR8^+^ Tregs secreted less cytokines than CCR8^-^ Tregs, whereas CCR8^+^ Teff secrete IL-4, but not IFN-*γ* and IL-17 (**Supplementary Figure 2**). Overall, our data showed that the secretome was able to impair the capacity to secrete Th-like cytokines, promote CCR8 expression and induce regulatory molecules. In order to evaluate whether the effect of the secretome was sustained over time after removing the cells from the malignant environment, we washed the cells after co-culture with secretomes, cultured them again in new media for 48h and analyzed phenotype and cytokine secretion. The results showed a significant reduction of Teff, but not Tregs after previous co-culture with malignant secretome (**Figure 4A**). CCR6 was upregulated in Teff previously co-cultured with OSCC secretome, whereas CCR8 maintained its up regulation in both subsets (**Figure 4B**). PD-1 and TIGIT also maintained their significant upregulation after previous co-culture with malignant secretome in both subsets (**Figure 4C**). No differences were observed in cytokine secretion between Tregs, however for Teff, IL-17 and IL-10 maintained its downregulation after removing the secretome (**Figure 4D**) but is difficult to interpret these results since Teff viability was compromised. Our results indicate that the OSCC secretome affects the viability of Teff after exposure, induces and sustained the up regulation of CCR8, PD-1 and TIGIT expression even after removing the secretome and suppresses cytokine production during direct contact.

### Transcriptomic Immune Characterization Revealed That Secretome From OSCC Potentiate the Vitamin D and Prostaglandin E Signaling in Tregs and Teff

**Figure 5:**
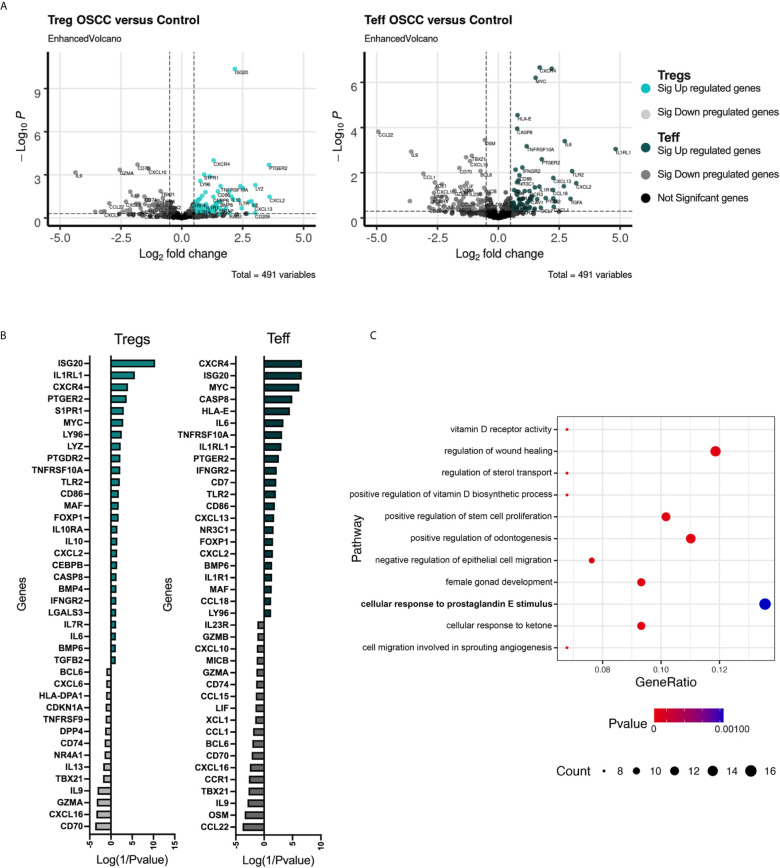
Transcriptomic analysis of Tregs and Teff after co-culture with control or OSCC secretome revealed pathways associated with the VitD and PGE2 signaling. **(A)** Volcano plots showing RNA-seq data obtained from 3 paired Tregs and Teff after co-culture with control or OSCC secretome. Vertical dotted lines indicate 1.5-fold change threshold and horizontal dotted line indicate P value 0.05. Colored dots show significant up regulated genes, whereas grey dots show significant down regulated genes in Th subsets when comparing cells co-cultured with OSCC secretome versus control secretomes. **(B)** Heatmap showing upregulated (colored with positive values) and downregulated (grey with negative values) genes in Tregs and Teff after co-culture with control or OSCC secretome. Log(1/Pvalue) was used to normalize the p values obtained when comparing each gene between control and OSCC secretome in Treg or Teff. **(C)** A pathway enrichment analysis was performed using the Gene Ontology Consortium database (data-version Released 2021-02-01) including biological processes. Cytoscape v.3.8.2 with the ClueGO plugin v.2.5.7 was used with a (p<0.01) and a kappa statistics score = 0.4 to calculate the relationships between the terms based on the similarity of their associated genes. Circles represent gene counts found in each pathway and p value is the probability of seeing at least x number of genes out of the total n genes in the list annotated to a particular GO term.

After demonstrating that the secretome is capable of affecting both Tregs and Teffs phenotypically and functionally, we analyzed the transcriptomic immune profile in 3 paired-donor peripheral blood Tregs and Teff from healthy volunteers after 48h of co-culture with OSCC or control secretome using the same protocol previously for **Figure 2**. After co-culture, cells were washed, stored in Trizol and 491 immune related genes were analyzed using the Human Inflammation and Immunity Transcriptome RNA targeted panel. We aim to identify relevant genes and potential pathways promoted or inhibited by the OSCC secretome in Th cells. We compared the transcriptome from Tregs and Teffs co-cultured with OSCC versus control secretome using volcano plots (**Figure 5A**). We then identified the top up regulated genes (positive value) and down regulated genes (negative value) according to their p value, normalized as Log(1/pvalue) in both subsets (**Figure 5B**). Results revealed that several transcripts were commonly upregulated in Tregs and Teff such as *ISG20, CXCR4, IL1RL1, PTGER2, MYC, CASP8, CD86, FOXP1, TLR2, CXCL2 and MAF*. Additionally, similar transcripts were commonly downregulated in Tregs and Teff such as *CD74, IL-9, TBX21* (Tbet), CXCL16, CD70 and GZMA (**Figure 5B**) (**Supplementary Table**). Interestingly, we did not observe significant differences regarding CCR8 expression, however we observed higher expression of its ligand CCL18 in Th cell co-cultured with OSCC secretome. After analyzing gene expression, we investigated significant signaling pathways found in Tregs and Teff co-cultured with OSCC by performing a pathway enrichment analysis using the Gene Ontology Consortium database (**Figure 5C**). The analysis revealed 11 significant pathways, from which the most related to T cells responses were associated with VitD signaling, wound healing regulation, prostaglandin E response, angiogenesis, negative regulation of epithelial cell migration, sterol transport and response to ketone. Other pathways identified were positive regulation of odontogenesis and female gonad development. VitD and PGE2 have been previously associated with CCR8 expression and Th1 inhibition, thus we evaluate the content of the secretome to see whether these metabolites were present.

### Proteomic Analysis of Secretome From Oral Cancer Revealed a Significant Pathway Associated With Prostaglandin E Production by the Vitamin D Membrane Cascade in OSCC

**Figure 6:**
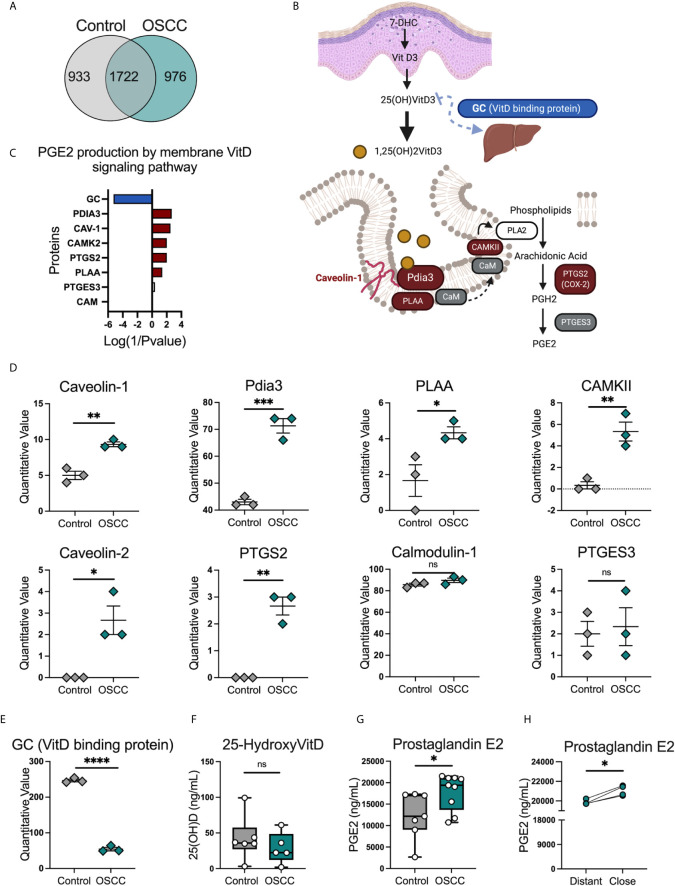
Proteomic analysis identified several proteins related with the PGE2 production by membrane vitamin D signaling pathway in OSCC secretomes. **(A)** Venn diagram of unique and common proteins identified in secretome obtained from biopsies from 5 OSCC and 5 control samples using timsTOF Pro.**(B)** Proteins and **(C)** diagram of the PGE2 production by membrane vitamin D signaling pathway. Briefly, overexpressed proteins in OSCC were colored in red, reduced proteins in OSCC were colored in blue and proteins present in the secretomes but with no statistical difference between control and OSCC were colored in grey. **(D)** Quantitative values of proteins from the PGE2 production by membrane vitamin D signaling pathway, data are presented as mean ± SEM using scatter dot plots (Unpaired t test). **(E)** Quantitative values of vitamin D binding protein or GC, data are presented as mean ± SEM using scatter dot plots (Unpaired t test). **(F)** Levels of 25(OH)VitD and **(G)** PGE2 were compared between cancer and control secretomes, data are presented as mean ± SEM using bars with scatter dot plots (Unpaired t test). **(H)** Levels of PGE2 were measured in secretomes from distant and close OSCC biopsies to the tumor site, data is presented with individual symbols with paired lines (Paired t test). For all statistical tests, ^∗∗∗∗^p < 0.0001, ^∗∗∗^p < 0.001, ^∗∗^p < 0.01 and ^∗^p < 0.05 were considered significant. ns, not significant.

The protein content of OSCC and control secretomes was evaluated in order to delineate the potential mechanisms associated with CCR8 expression and the pathways observed in the transcriptomic analysis. A qualitative and quantitative proteomic analysis was performed in 5 OSCC and 5 non-malignant pooled secretomes. The data revealed that 976 proteins were found exclusively in cancer secretome, 933 proteins were found exclusively in control secretome and 1722 proteins were found in both conditions (**Figure 6A**)(**Supplementary Table**). Scaffold4.0 and intuitive pathway analysis (IPA) were used to analyze the data set in a quantitative manner. The analysis revealed amongst diseases associated with OSCC secretomes were; cancer, connective tissue disorders and infectious diseases, (**Supplementary Table**). Looking at relevant groups of proteins differentially expressed between samples, we observed enrichment of proteins from the PGE2 production by rapid membrane VitD signaling pathway (**Figures 6B, C**), including Pdia3, Caveolin-1, PLAA, CAMKII and PTGS2 (**Figure 6D**). Interestingly, Pdia3 has been previously reported as one of the key hub genes in OSCC, validated by gene expression and immunohistochemistry (28). Within the VitD pathway, the VitD binding protein (VDBP also known as GC) was significantly reduced in OSCC samples (**Figure 6E**), suggesting an impairment in the transport of VitD from the skin to circulation as previously reported (29), which suggest that this metabolite is more concentrated in cancer samples. In order to understand whether the VitD rapid signaling pathways was associated with the Th phenotypic and functional changes, the levels of 25-hydroxyvitamin (25(OH)) VitD and PGE2 were measured in the secretomes. First, we observed similar levels of VDBP-unbound 25(OH) VitD (**Figure 6F**) in both conditions, however PGE2 was higher in OSCC samples than control samples (**Figure 6G**) and in samples obtained from cancer areas compared with samples obtained from distal cancer areas from the same cancer patient (**Figure 6H**). This data suggested that the production of VitD *in vitro* is not different as the same amount of tissue was used in culture. Despite this, the induction of PGE2 was augmented in cancer secretomes suggesting that this signaling pathways is activated in OSCC. In addition, the GC (VitD binging protein) was one of the top ten significantly reduced proteins in the OSCC proteomic analysis, suggesting that the transport of VitD from the tissue to peripheral circulation may be impaired, inducing an accumulation of VitD in the malignant environment. Overall, the characterization of the OSCC secretome revealed several proteins associated with the prostaglandin E production by rapid membrane VitD signaling and potential accumulation of VitD by reduced presence of the VitD binding protein.

### VitD Promote a Th2-like Treg Phenotype and Combination of PGE2 and VitD Modulate CCR8 Expression and Cytokine Production in Th Cells

**Figure 7:**
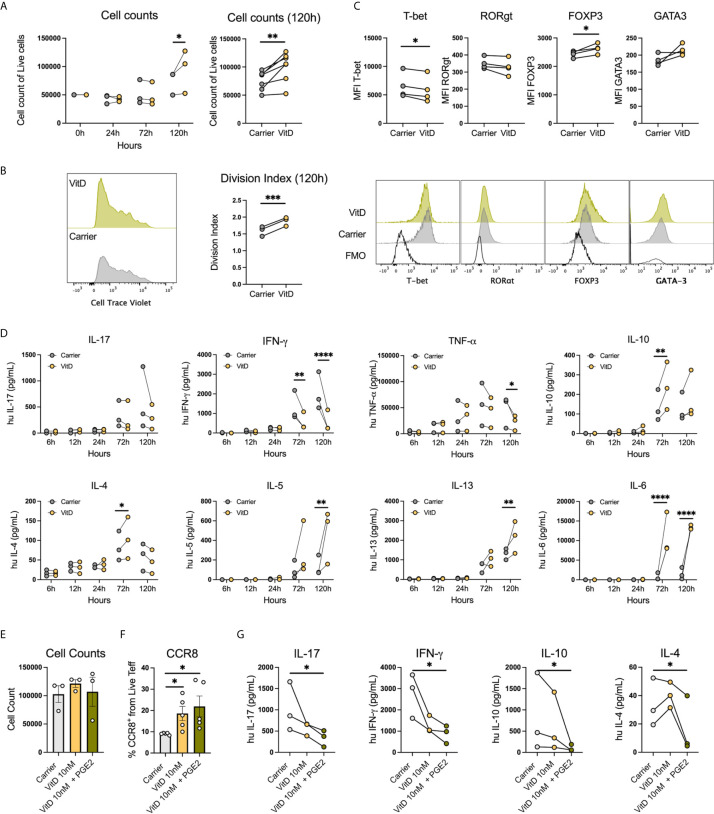
PGE2 with VitD induce CCR8 expression and inhibit cytokine production in Th cells. **(A)** Representative histograms and cumulative data of cell counts and **(B)** division index of sorted memory Teffs (2×10^5^) activated with anti-CD3/CD28 beads (1:5) in the presence or absence of 1,25(OH)VitD3 (10nM in ethanol) or Carrier (ethanol) at 24h, 72h and 120h post activation. Data are presented as individual symbols with paired lines (Two-way Repeated Measure ANOVA and Paired t test). **(C)** Representative histograms and cumulative data of transcription factor expression of sorted memory Teffs (2×10^5^) activated with anti-CD3/CD28 beads (1:5) in the presence or absence of VitD (10nM in ethanol) or Carrier (ethanol) at 120h post activation. Data are presented as individual symbols with paired lines (Paired t test). **(D)** Cytokines were measured in supernatants obtained from sorted memory Teffs (2×10^5^) activated with anti-CD3/CD28 beads (1:5) in the presence or absence of 1,25(OH)VitD3 (10nM in ethanol) or Carrier (ethanol) at 6h, 12h, 24h, 72h and 120h post activation. Data are presented as individual symbols with paired lines (Two-way Repeated Measure ANOVA). **(E)** Cell counts, **(F)** CCR8 expression and **(G)** cytokine production were measured in anti-CD3/CD28 pre-activated Teff cells (1x10^5^) cocultured with carrier (ethanol), 1,25(OH)VitD3 (10nM in ethanol) or 1,25(OH)VitD3 (10nM in ethanol) in combination with PGE2 (5uM) for 72h with flow cytometry. Data is presented as mean ± SEM using column bars plots with bars with scatter dot plots for phenotype and individual symbols with paired lines values for cytokine production (Paired t test). For all statistical tests, ^∗∗∗∗^p < 0.0001, ^∗∗∗^p < 0.001, ^∗∗^p < 0.01 and ^∗^p < 0.05 were considered significant.

Since VitD and PGE2 were within the pathways identified in the transcriptomic and proteomic analysis, we evaluated whether these metabolites were associated with the changes induced by OSCC secretome. First, we analyzed cell counts of live sorted memory Teff from peripheral blood after anti-CD3/CD28 activation in the presence or absence of VitD (10nM) at 24h, 72h and 120h post-activation, as it has been shown that VitD has antiproliferative properties (30). Our data showed that cell counts (**Figure 7A**) and division index (**Figure 7B**) were significantly higher in the presence of VitD after 5 days. We then analyze whether VitD also modulates Th transcription factors at 120h post activation in the presence or absence of VitD (10nM) and observed significant inhibition of Tbet and induction of FoxP3 in the presence of VitD (**Figure 7C**). We next characterized the secretion of Th cytokines on sorted memory Teff from peripheral blood following anti-CD3/CD28 activation in the presence or absence of VitD (10nM) at 6h, 12h, 24h, 72h and 120h post-activation. The VitD receptor is induced after TCR activation (31), thus, we observed significant differences at 72h and 120h post-activation in response to VitD (**Figure 7D**). The data showed that VitD inhibits Th1 responses by significantly reducing IFN-*γ* and TNF-α production, limits IL-17 secretion and promotes IL-10 and Th2 cytokines such as IL-4, IL-5, IL-6 and IL-13. We then analyzed the effect of PGE2 in combination with VitD in pre-activated Teff for 72h and we observed no difference in cell counts (**Figure 7E**), however both VitD and PGE2 induced CCR8 expression (**Figure 7F**). When cytokine secretion was analyzed, we observed that PGE2 inhibited secretion of IFN-*γ*, IL-17, IL-10 and IL-4 (**Figure 7G**). Altogether these results demonstrated that VitD modulates Th responses by causing an imbalance in the Th1/Th2 responses and by inducing regulatory cells by promoting FoxP3 expression. In addition, VitD and PGE induce CCR8 expression and inhibit cytokine secretion.

### CCR8 Ligand CCL18 Is Increased in Histological Samples From Malignant Oral Mucosa and Promote CCR8 Upregulation by Direct Contact

**Figure 8:**
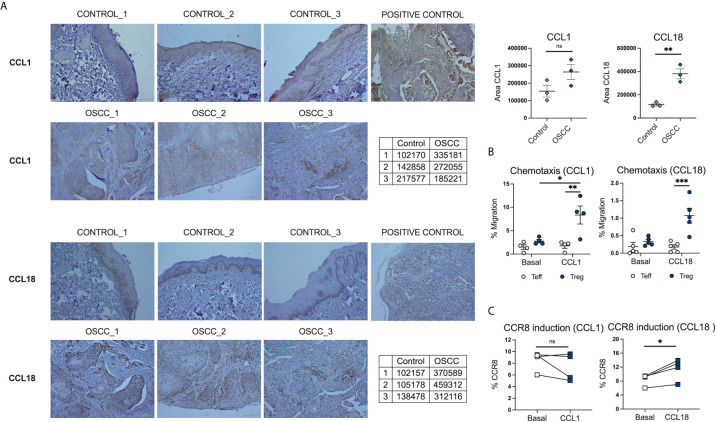
CCL18 is augmented in histological samples of OSCC patients and also induce CCR8 expression in Teff. **(A)** Representative histological staining of CCL1 and CCL18 in a biopsy from a patient with OSCC and a patient without malignancy, using colon carcinoma as a positive control for CCL1 and melanoma as a positive control for CCL18. **(B)** Semi-quantification of area for CCL1 and CCL18 staining by ImageJ, data is presented as mean ± SEM using individual values described in the tables (Unpaired t test). **(C)** Percentage of migrated memory Teffs and Tregs to recombinant chemokines CCL1 and CCL18. Sorted Cell trace violet^+^ Memory Teffs (5×10^4^) and unstained memory Tregs (5×10^4^) were placed in the top chamber of a 5-μm-pore Transwell filter system. Bottom chambers were filled with media only, CCL1 or CCL18, (all 0.5 ug/mL). The percentage of migration for each subset was calculated as (number of cells in the bottom chamber after 1 h × 100)/initial number of cells in the top chamber. Data are presented as mean ± SEM using scatter dot plots (Paired t test). **(C)** CCR8 expression was measured in pre-activated memory Teffs (1x10^5^) cultured with media only, or media with CCL1 or CCL18, (all 0.5 ug/mL) for 72h, data are presented using individual symbols with paired lines (Paired t test). For all statistical tests, ^∗∗∗^p < 0.001, ^∗∗^p < 0.01 and ^∗^p < 0.05 were considered significant. ns, not significant.

Beside the role of skin mediations (32) in the induction of CCR8 expression, the effect of their ligands CCL1 and CCL18 (33) has also been associated with the upregulation of its receptor and chemotaxis of CCR8^+^ cells. Thus, we analyzed the expression CCL1 and CCL18 in OSCC and control histological samples. The analysis revealed that CCR8 ligands, CCL1 and CCL18, were highly expressed in the oral cavity, however only CCL18 reach significance when comparing OSCC tissues with non-malignant oral mucosa (**Figure 8A**) (**Supplementary Figure 3**). Interestingly, the expression of CCL18 was mainly observed in the basal stratified squamous epithelium in non-malignant samples, whereas its expression in cancer samples was within the squamous cell carcinoma. CCL1 and CCL18 may either play a role in CCR8^+^ Treg migration to the malignant zone of oral cancer or they might induce its expression directly, thus, we measure chemotaxis and CCR8 induction in response to recombinant chemokines CCL1 and CCL18. Peripheral blood Tregs and Teff were isolated from the same donor, Teff were stained with Cell trace violet and both subsets were combined in a 1:1 ratio and seeded in the top chamber of a 5um Transwell. In the bottom chamber recombinant chemokines CCL1 or CCL18 were added and media without chemokines was used as a control. After 1h, migrated cells were recovered and counted (**Supplementary Figure 4**). When T cell migration was analyzed, we observed that CCL1 and CCL18 induce preferential migration of Tregs over Teff, however only migration to CCL1 induce significant chemotaxis in comparison with media without chemokines (**Figure 8B**). When the effect of direct contact was analyzed, we observed that only CCL18 induced CCR8 expression in pre-activated Teff (**Figure 8C**). This data showed that CCL18 is increased in OSCC and it can also induce CCR8 expression independently of the VitD signaling pathway.

## Discussion

T lymphocytes have been the most representative and well-studied tumor-infiltrating subset in oral cancer. The presence of infiltrated Th cells in tumors has been correlated with rapid cancer progression (34) and poor prognosis (35). Several studies have identified the phenotype of Th cells in tumors and some authors have observed an imbalance in the different Th lineages in oral cancer, being Th2 cells augmented and Th1 cells reduced in comparison with samples from healthy donors (36). In general, pro-inflammatory Th1 responses have been associated with good prognosis in cancer, as these responses increase macrophage mediated phagocytosis, activates B cells to promote the production of opsonizing antibodies, activates complement and activates CD8^+^ T cells to promote cytotoxic mechanisms (37). Th2 cytokines,such as IL-4 and IL-10, are increased in late-stage cancers in comparison to Th1 cytokines that are more prevalent in the early-stage (38). This indicates that the immune responses are associated with cancer progression, and changes in the repertoire of cells directed by the tumor could be detrimental. Th17 cells have also been associated with tumor progression in oral cancer (39), as well as Tregs, which have been found increased not only in the oral tissue, but also in peripheral blood (40, 41), expressing CTLA-4^+^, HLA-DR^+^ and granzyme B^+^ (42) and inhibiting IFN-γ, and promoting IL-10 and TGF-β secretion (43, 44). In addition, a positive correlation between Treg infiltration and the TNM score has been observed in this cancer (45, 46). Furthermore, T cells can modulate other immune cells such as macrophages, which can also potentiate cancer progression, specially M2 macrophages, as previously reported (47).

Our previous data reveled a significant association between Th2-like Tregs with colorectal cancer and melanoma, however despite the fact we observed higher percentages of Th2-like Tregs in OSCC in comparison with control samples, Th-like subsets overall did not cover the majority of the memory Treg population as it occurs in peripheral blood due to high CCR4 expression in circulation (15). This was an interesting observation as CCR4 has been previously used to identify cutaneous Th subsets (48). On the other hand, CCR8 was the main chemokine receptor expressed in Tregs from breast cancer (49, 50), and in Tregs of lung adenocarcinoma, melanoma and colorectal adenocarcinoma in comparison with their counterpart (51) effector population (49). CCR8 is also increased in Tregs from colorectal cancer (51). In term of the role of CCR8 in Tregs, Coghill et al. demonstrated in a graft-versus-host disease (GVHD) mouse model wherein CCR8 was required for Treg survival *in vivo*. Interestingly, this study showed no effect in terms of activation and proliferation and the addition of CCL1 and CCL18 showed no effect on Treg viability *in vitro*. However they suggest that the interaction between Tregs and DCs was required to induce CCR8-mediated survival (52). Other studies analyzed CCR8 Tregs from human blood analyzed their suppressive capacity in the presence of four CCR8 ligands CCL1, CCL18, CCL16 and CCL18. Their results demonstrated that CCL1 was the only ligand able to promote Treg suppressive function and Ca2^+^ flux post activation (33). However, previous data from other authors demonstrated that CCL18 was also able to induce Ca2^+^ flux in CCR8 transfected cells (53). With regards to CCR8 expression, Barsheshet et al. (33 *)*, showed increased CCL1-mediated CCR8 expression in Tregs, however they did not analyze the effect of other ligands in order to understand whether this effect was specific to CCL1. Our data showed that CCL18 was the main chemokine increased in OSCC, associated to cancer cells mainly by immunohistochemical analysis. This could be explained by the important role of CCL18 in oral cancer where it promotes hyperplasia and metastasis by JAK2/STAT3 signaling pathways (54). In fact, in a study focused on the alterations of chemokine and chemokine receptors in premalignant stages of OSCC, CCL18 was the top one gene significantly upregulated in oral leukoplakia samples in comparison with normal epithelia (55). In this context, another study demonstrated that CCL18 induced cell epithelial-mesenchymal transition and promoted cell migration and invasion (56), therefore it would be interesting to investigate factors that promote CCL18 expression in oral epithelia, and how CCL18 upregulation affects cancer cells. We observed that the OSCC secretome induced *CCL18* gene expression in Teff and CCL18 was able to promote CCR8 expression, therefore it would be interesting to observe how the tumor environment is able to regulate this chemokine to promote CCR8^+^ cells. We demonstrated that CCR8^+^ Teff were reducing Th1 responses and promoting Th2 responses, whereas CCR8^+^ Tregs produce less cytokines than CCR8^-^ Tregs. This result suggests that CCR8 expression in Tregs identifies a population with a reduced capacity to secrete cytokines, both anti and pro inflammatory. Since the transcriptomic data showed induction of IL-10 in Th cells by OSCC secretome, it is possible that other post transcriptional mechanisms may play a role in the regulation of surface markers and cytokines.

VitD signaling responses can be triggered by gene transcription after VitD-VitD Receptor (VDR) binding to response elements and by Pdia3-mediated rapid membrane response (57). The latter is a rapid response that requires the presence of Pdia3 and Cav-1, where Cav-1 acts as a scaffolding protein, and Pdia3-Cav-1 form a membrane receptor complex in caveolae, triggering the binding of PLAA to Pdia3 and activating PLA2 *via* PLAA (57, 58). Subsequently the activation of PLA2 by PLAA, results in the production of PGE2 *via* arachidonic acid (58). A largescale transcriptomics analysis of differentially expressed genes from 326 OSCC and 165 normal controls revealed that the main enriched pathway regulated were extracellular matrix (ECM)-receptor interaction and focal adhesion according to several genes related to ECM structure such as laminins, collagen and integrins (28). The authors also revealed three upregulated hubs (defined as genes with significant interaction partners regulating the differentially expressed genes), named BGH3 (Transforming growth factor-beta-induced protein ig-h3), MMP9 (Matrix metalloproteinase-9) and Pdia3. The hub genes were then validated by immunohistochemistry and Pdia3 was absent in normal oral mucosa, while a high percentage of positive expression was found in OSCC (28). In addition, Pdia3 in combination with caveolin and PLAA, have been associated with the production of PGE2 by VitD signaling (57, 58), which in turn is associated with CCR8 expression on T cells (32). Our proteomic analysis showed MMP9 and Pdia3 as proteins significantly increased in OSCC samples. In addition, several proteins related with the rapid membrane VitD pathway were upregulated. The data also revealed reduced VDBP in OSCC proteomic samples, suggesting a potential imbalance in the transport of VitD, promoting its accumulation in the cancer area,as previously shown in a VDBP knock out mouse model (29). Our transcriptomic data showed several pathways associated with VitD and PGE2 responses, possibly due to the accumulation of VitD in cancer areas and the production of PGE2 *via* VitD membrane signaling pathway. In addition, one of the top genes up regulated by the secretomes in both T cell subsets was PTGER2, the prostaglandin E receptor 2, suggesting that the secretome not only contained more PGE2, but also induce the transcription of the receptor, possibly due to the effect of PGE2 (59). PGE2 as well as VitD has been shown to inhibit Th1 responses (60, 61), which was one of the main effects of the OSCC secretome by downregulating Tbet (*TBX21*) and IFN-*γ* production. These results suggest that the cancer impairs the VitD transport, promoting VitD accumulation and the activation of the production of PGE2 *via* the VitD membrane signaling pathway. In this environment, activated T cells expressing VitD receptor respond to these metabolites by reducing antitumor responses and promoting a regulatory phenotype.

It is well known that the TME can support angiogenesis, tumor progression, and immune evasion from T lymphocyte recognition (62). In this context, the immune checkpoint (e.g., PD-1, PD-L1, or TIGIT), can be modified by the TME to impair the endogenous antitumor T cell responses (62). Interestingly, high PD-L1 expression has been associated with good overall survival in oral squamous cell carcinoma (12), however other authors have shown increased PD-1-PDL-1 expression by conventional and fluorescent immunohistochemistry in OSCC, even before malignant transformation in early premalignant lesions (63). Other studies found an association between PD-L1 and PD-1 immunoreactivity and malignant clinicopathological features and a poor prognosis (64, 65). We did not check PD-1 or PDL-1 expression in tissues, but we found that OSCC secretomes were able to upregulate PD-1 expression on Teff and Tregs. The induction of PD-1 in T cells can promote PD-1-PDL-1 binding, which in turn inhibits the lymphocytes activation and cytokine secretion (66). TIGIT is another inhibitory molecule that has been found in several studies aimed at identifying genetic profile of tumor infiltrating T cells. This marker is associated with inhibition of Th1 and Th17 responses, but not Th2 responses (27). In cancer, co-expression of TIGIT and PD-1 has been observed in tumor infiltrated CD8^+^ T cells (67) and its expression is increased in Tregs within Th subsets (68). CD155, expressed in cancer cells, binds to TIGIT on T cells to induce direct inhibitory signals and disrupt CD226-mediated T cell activation (69). Interestingly, we observed no induction of PD-1 and TIGIT by VitD, thus these markers were induced by other unknown mechanisms.

Traditionally, OSCC has been associated with late-stage diagnosis and poor prognosis. Palliative care is the only treatment in some cases, and when surgery is possible, it can prolong survival, but it also affects the quality of life of the patients and their relatives. It is thus crucial to understand the molecular aspects of this cancer in order to identify potential mechanism to improve the anti-tumor response. This study revealed novel information regarding the immunoregulatory effect of tumor environment from OSCC affecting Th subsets. The understanding of these responses could help to identify potential treatments in order to improve survival in patients with late-stage OSCC.

## Data Availability Statement

The datasets presented in this study can be found in online repositories. The names of the repository/repositories and accession number(s) can be found in the article/**Supplementary Material**.

## Ethics Statement 

The studies involving human participants were reviewed and approved by Ethical Committee from the Health Service Talcahuano number 19-06-11 and Ethical Committee from the Health Service Concepcion number 19-03-07. The patients/participants provided their written informed consent to participate in this study.

## Author Contributions

MF, FL, and EN-L performed experiments. MY and MS recruited patients, analyzed histological samples, defined OSCC pathological state, participated in the OSCC diagnosis and collected the clinical data. MH designed, performed, and analyzed the proteomic data. GN analyzed the proteomic data. EN-L, MF, GN, FZ, GL, and FA performed the RNA sequencing experiment. LL analyzed vitamin D levels. MY, MS, JU, LN, SC, OZ, AC, LC, SM, JP, MN, JG, JF, and RP performed surgery or dental procedures resulting in sample collection. CR and CS contributed with pathway analysis from proteomic data. GC performed cell sorting. MV analyzed the transcriptomic data. MF and EN-L wrote the first draft of the manuscript. EN-L and RM wrote the second draft of the manuscript. MF and EN-L organized the database, analyzed the data, performed pathway analysis, transported the samples, created the figures and performed the statistical analysis. EN-L designed the study and directed the project. All authors contributed to the article and approved the submitted version.

## Funding

This research was funded by the Chilean Agency of Investigation and Development (ANID) grant FONDECYT 11170610 and PAI79170073. MF was funded by FONDECYT 11170610, Sindicato-2 Postgraduate Scholarship and University of Concepcion Postgraduate Scholarship. FL was funded by University of Concepcion Postgraduate Scholarship. EN-L was funded by FONDECYT 11170610, PAI79170073 and FONDECYT 1211480. CS is supported by Lions Medical Research Foundation, Diabetes Australia, National Health and Medical Research Council (NHMRC) and FONDECYT 1170809.

## Conflict of Interest

The authors declare that the research was conducted in the absence of any commercial or financial relationships that could be construed as a potential conflict of interest.
