# Supplementary material for: Immunomodulation of T Helper Cells by Tumor Microenvironment in Oral Cancer Is Associated With CCR8 Expression and Rapid Membrane Vitamin D Signaling Pathway
Source: Front Immunol. 2021 May 7;12:643298. doi: 10.3389/fimmu.2021.643298 (PMC8137990; doi:10.3389/fimmu.2021.643298)
Supplement: Supplementary file 1 [file Presentation_1.pdf]

Supplementary Material

Immunomodulation of T helper cells by tumor microenvironment in oral cancer is associated with CCR8 expression and rapid membrane Vitamin D signaling pathway

Marco Fraga[1], Milly Yáñez[2], Macarena Sherman[3,4,5], Faryd Llerena[1], Mauricio Hernandez[6], Guillermo Nourdin[6], Francisco Álvarez[6], Joaquín Urrizola[7] César Rivera[8], Liliana Lamperti[1,9], Lorena Nova[10], Silvia Castro[1], Omar Zambrano[11], Alejandro Cifuentes[11], León Campos[12], Sergio Moya[12], Juan Pastor[12], Marcelo Nuñez[12], Jorge Gatica[12], Jorge Figueroa[12], Felipe Zúñiga[1], Carlos Salomón[13], Gustavo Cerda[14], Ricardo Puentes[5], Gonzalo Labarca[1], Mabel Vidal[15], Reuben McGregor[16] and Estefania Nova-Lamperti[1*]

*** Correspondence:**
Corresponding Author
enovalamperti@gmail.com, enova@udec.cl

Project Accession

The mass spectrometry proteomics data have been deposited to the ProteomeXchange Consortium via the PRIDE partner repository with the dataset identifier PXD023049. The accession number for the RNA-seq is GSE171638.

1.1 Supplementary Figures

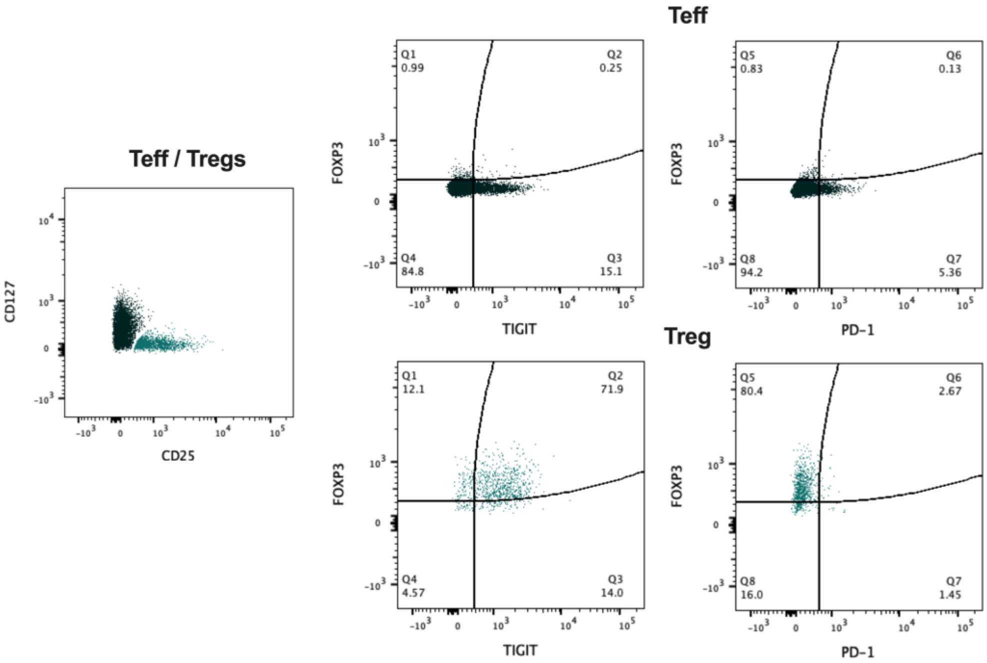

Supplementary Figure 1. FOXP3 expression on CD127^{low}CD25^{hi} Tregs. CD4^{+}T cells were divided into Teff and Tregs based on CD127 and CD25 expression. FOXP3 expression was confirmed on CD127^{low}CD25^{hi} Tregs and Teff.

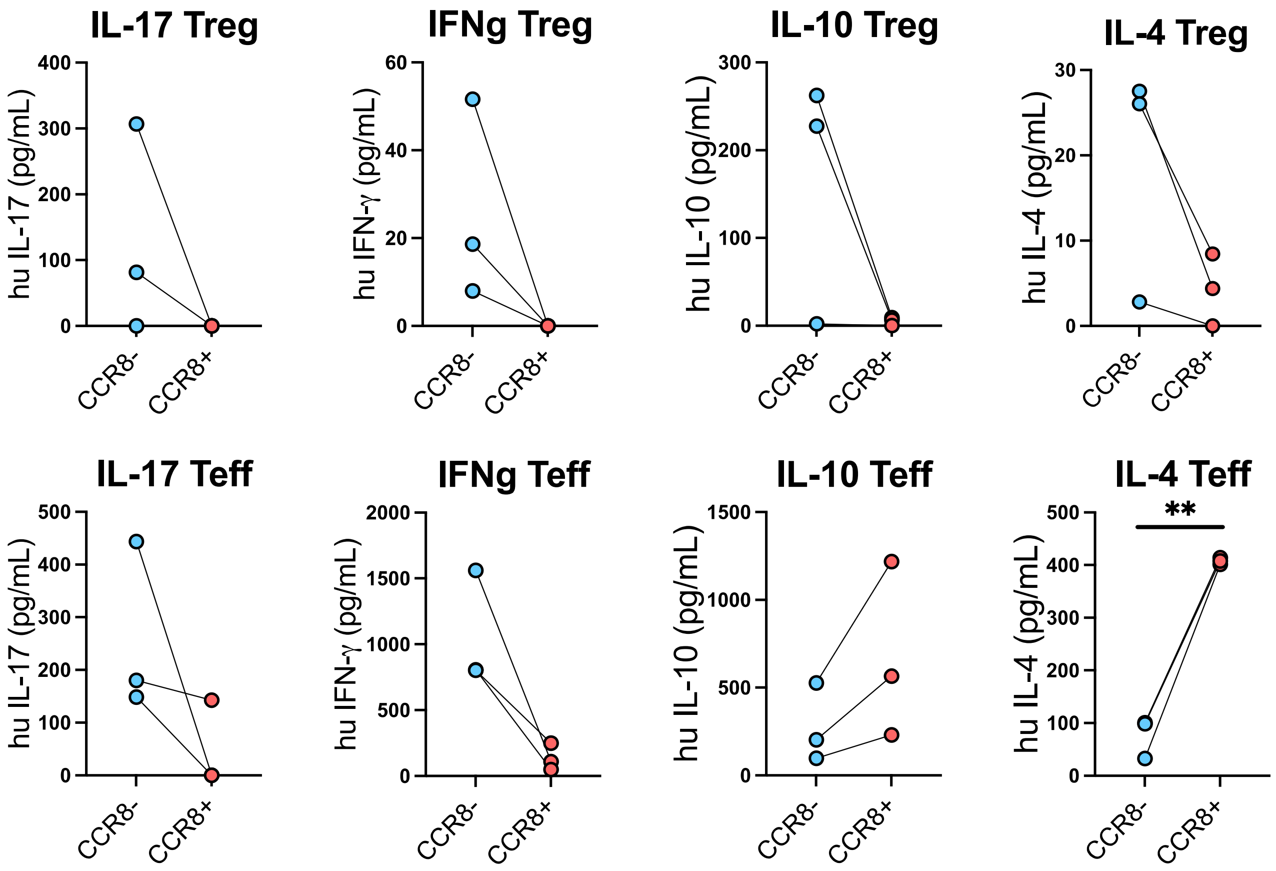

Supplementary Figure 2. Analysis of cytokine production by $CCR8^+$ and $CCR8^-$ Treg and Teff. Sorted $CD4^+$ T cells were sorted as Treg $CCR8^+$, Treg $CCR8^-$, Teff $CCR8^+$ and Teff $CCR8^-$. $1x10^5$ cells were activated with anti-CD3CD28 beads (1:5) and cytokines in the supernatant were analyzed with Th1/2/17 cytokine bead array kit. Data are presented using individual symbols with paired lines (Paired t test).

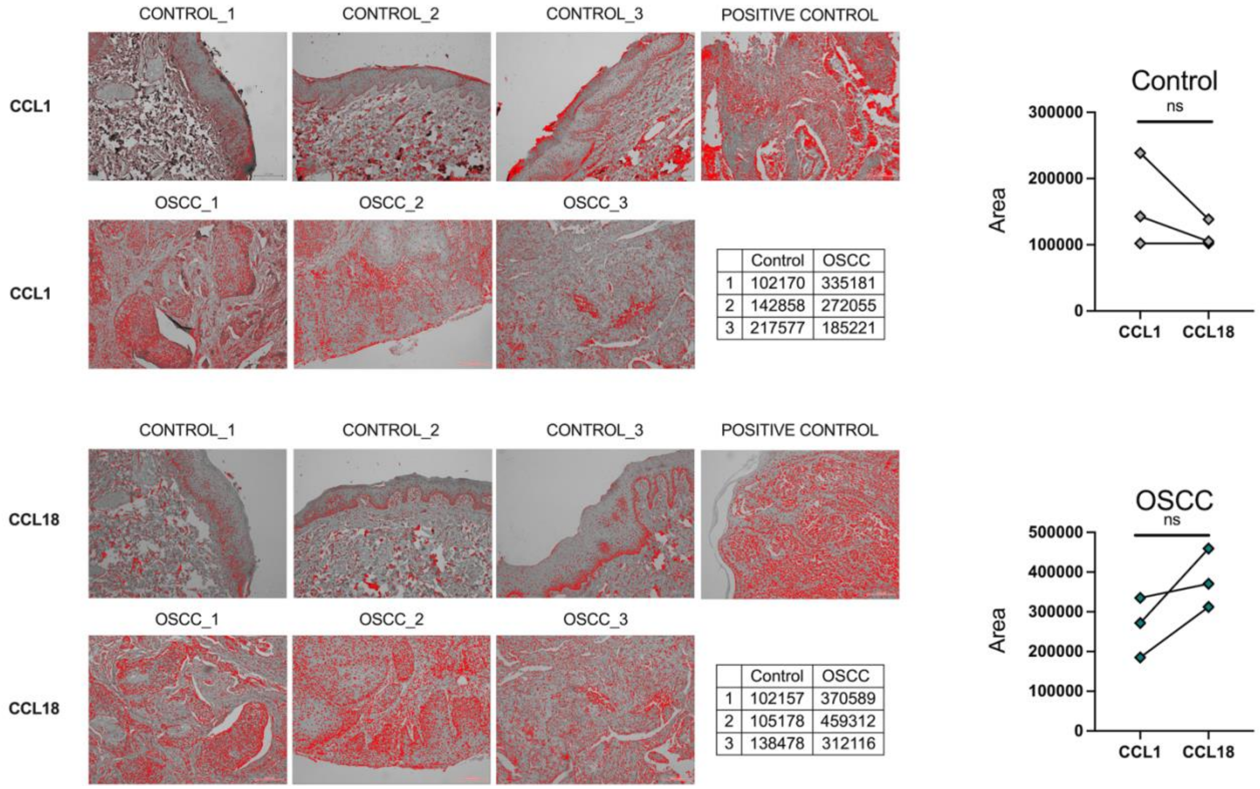

Supplementary Figure 3. Immunohistochemical analysis (in red) of CCL1 and CCL18 in biopsies from 3 OSCC and 3 control samples, using colon carcinoma as a positive control for CCL1 and melanoma as a positive control for CCL18.

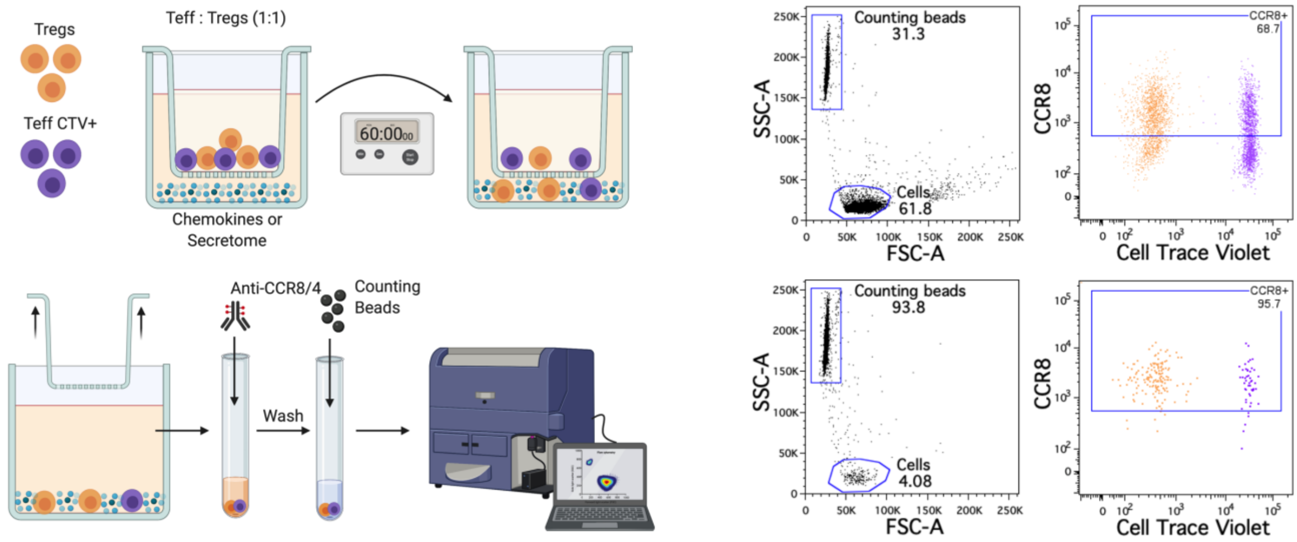

Supplementary Figure 4. Representative dot plot example of migratory assays. Sorted cell trace violet+ Memory Teffs (5×10^4) and memory Tregs (5×10^4) were placed in the top chamber of a 5-µm-pore Transwell filter system. Bottom chambers were filled with control or cancer secretome and media only or CCL18, CCL17, CCL22, CXCL10 (all 0.5 ug/mL). Teff and Tregs were quantified after 1h by flow cytometry. Data was acquired on FortessaX20 and analyzed with FlowJo.

2 Supplementary Materials

Reagents	Catalogue	Company
Donkey Anti-Rabbit IgG H&L (HRP)	ab97064	Abcam
Lymphoprep	04-03-9391/02	Axis Shields
BD Cytometric Bead Array (CBA) Human Th1/Th2/Th17 Cytokine Kit	560484	BD Biosciences
CCL1/TCA3 antibody	orb13280	Biorbyt
MIP4/CCL18 antibody	orb13576	Biorbyt
Recombinant Human CCL1 (I-309) (carrier-free)	582702	BioLegend
Alexa Fluor 488 anti-human CD279 (PD-1) Antibody	367407	BioLegend
Alexa Fluor 647 anti-human TIGIT (VSTM3) Antibody	372723	BioLegend
PE anti-human CD198 (CCR8) Antibody	360604	BioLegend
Brilliant Violet 421 anti-human CD4 Antibody	317434	BioLegend
Brilliant Violet 650 anti-human CD196 (CCR6) Antibody	353426	BioLegend
APC/Cyanine7 anti-human CD45RA Antibody	304128	BioLegend
PE/Cyanine7 anti-human CD25 Antibody	356108	BioLegend
FITC anti-human CD183 (CXCR3) Antibody	353704	BioLegend
APC anti-human CD194 (CCR4) Antibody	359408	BioLegend
PerCP/Cyanine5.5 anti-human CD4 Antibody	317428	BioLegend
APC anti-human CD127 (IL-7Rα) Antibody	351315	BioLegend
Pacific Blue anti-human FoxP3 Antibody	320216	BioLegend
Alexa Fluor® 647 anti-GATA3	653809	BioLegend
HTS Transwell 96-well Permeable Support	3388	Corning
CD127 Monoclonal Antibody (eBioRDR5) PerCP-Cyanine5.5	45-1278-42	eBiosciences
FoxP3/Transcription Factor Staining Buffer	00-5523-00	eBiosciences
Anti-Human/Mouse ROR gamma (t) APC	17-6988-80	eBiosciences
eFluor 450 anti-human FoxP3 Antibody	48-4777-42	eBiosciences
T-bet Monoclonal Antibody (eBio4B10 (4B10)), PerCP-Cy5.5	45-5825-82	eBiosciences
PGE2 high sensitivity ELISA kit	ADI-930-001	Enzo
1α,25-Dihydroxyvitamin D3	BML-DM200-1000	Enzo
96 Well U-Bottom	353077	Falcon
Collagenase Type 1	17100-017	Gibco
PBS Tablets	18912-014	Gibco
Dynabeads Human T-Activator CD3/CD28 for T Cell Expansion and Activation	11131D	Gibco
Pen Strep Glutamine (100X)	10378-016	Gibco
NextSeq 500/550 Mild Output Kit v2.5	20024904	Illumina
CountBright Absolute Counting Beads	C36950	Invitrogen
Tissue Culture Plate 48 well	TCP 011048	Jet BIOFIL
LIVE/DEAD® Fixable Near-IR Dead Cell Stain Kit	L10119	Life Technologies
CellTraceTM Violet Cell Proliferation Kit	C34557	Life Technologies
CountBrightTM Absolute Counting Beads	C36950	Life Technologies
X-VIVO 15 Chemically Defined, Serum-free Medium	04-744Q	Lonza
Memory CD4+ T Cell Isolation Kit, human	130-091-893	Miltenyi Biotec
Proleukin recombinant IL-2	PL-00101/0936	Novartis
Recombinant Human CCL18/PARC Protein	NBP2-35041	Novus Biologicals
The Human Inflammation & Immunity Transcriptome RNA targeted panel 12-Index	RHS-005Z 333114	QIAgen
CORNING HTS TRANSWELL-96W	CLS3388-2EA	Sigma-Aldrich
DNase I	LS002140	Worthington
